# Antibiotic Resistance and Pathogenomics of *Staphylococci* Circulating in Novosibirsk, Russia

**DOI:** 10.3390/microorganisms9122487

**Published:** 2021-11-30

**Authors:** Alevtina Bardasheva, Artem Tikunov, Yuliya Kozlova, Elena Zhirakovskaia, Valeriya Fedorets, Natalya Fomenko, Tatyana Kalymbetova, Svetlana Chretien, Vitaliy Pavlov, Nina Tikunova, Vera Morozova

**Affiliations:** 1 Institute of Chemical Biology and Fundamental Medicine SB RAS, Lavrentiev Avenue 8, 630090 Novosibirsk, Russia; herba12@mail.ru (A.B.); arttik@ngs.ru (A.T.); ulona@ngs.ru (Y.K.); ezhr@niboch.nsc.ru (E.Z.); f.valeriya41@gmail.com (V.F.); 2 Joint-Stock Company Vector-Best, P.O. Box 121, 630117 Novosibirsk, Russia; FomenkoN@vector-best.ru (N.F.); Kalymbetova@vector-best.ru (T.K.); 3 Novosibirsk Research Institute of Traumatology and Orthopedics n.a. Ya. L. Tsivyan, Frunze 17, 630091 Novosibirsk, Russia; ssonovo@inbox.ru (S.C.); pavlovdoc@mail.ru (V.P.)

**Keywords:** *Staphylococcus*, coagulase-negative staphylococci, MDR, MRS, ST, pathogenomics, virulence factors, antibiotic resistance genes, Siberia, Russia

## Abstract

A total of 394 strains of staphylococci found in humans and pets in Novosibirsk, Siberian Russia, were characterized in terms of antibiotic resistance and corresponding genes. Two coagulase-positive and 17 coagulase-negative species were identified. The majority of isolates, with the exception of *S. haemolyticus* and hospital *S. epidermidis* isolates, were sensitive to most of the tested antibiotics, and isolates from pets displayed the lowest level of resistance. Nevertheless, methicillin-resistant (MRS) and/or multidrug-resistant (MDR) isolates were found in all prevailed species, including coagulase-negative. A set of genes corresponding to the detected resistance was identified: *mecA* (beta-lactam resistance), *aac(6′)-Ie-aph(2″)-Ia, aph(3′)-IIIa,* *ant(4′)-Ia* (aminoglycoside-modifying enzymes), *ermA/ermC*, and *msrA* (macrolide resistance). Complete genome analysis for ten MDR *S. epidermidis* and five MDR *S. haemolyticus* isolates revealed additional antibiotic resistance genes *mphC*, *qacA/qacB*, *norA*, *dfrC/dfrG*, *lnuA*, *BseSR*, and *fosB*. *NorA*, *dfrC*, and *fosB* were present in all *S. epidermidis* genomes, whereas *mphC* and *msrA* were identified in all *S. haemolyticus* ones. All investigated MDR *S. epidermidis* and four of five *S. haemolyticus* strains were moderate or strong biofilm producers, whereas multiple genes responsible for this function and for virulence and pathogenicity were identified mostly in *S. epidermidis*, but were less frequently represented in *S. haemolyticus*.

## 1. Introduction

Staphylococci are Gram-positive facultative anaerobic bacteria that belong to the *Staphylococcaceae* family and *Staphylococcus* genus. To date, approximately 60 validated members of the genus have been described (https://lpsn.dsmz.de/genus/staphylococcus, accessed on 10 September 2021). Most bacteria from this genus normally inhabit the skin and mucosae of humans and animals and are also a part of soil microbial communities. At the same time, staphylococci can affect almost any organs and tissues of the human body, causing superficial and deep purulent abscesses, respiratory and urinary tract infections, purulent–necrotic processes in postoperative wounds, and food poisoning or intoxication [1].

Staphylococci are divided into coagulase-positive (able to produce coagulase) and coagulase-negative species. Among coagulase-positive species, *Staphylococcus aureus* is the most clinically important. A number of other coagulase-positive staphylococci (*Staphylococcus delphini*, *Staphylococcus intermedius*, and *Staphylococcus pseudintermedius*, belonging to the *Staphylococcus intermedius* group) are causative agents of the most common staphylococcal infections in veterinary medicine and can also be transmitted to humans through close contact with animals [2,3,4]. Coagulase-negative members of the genus are considered less virulent and are usually identified as commensals [1]. Nevertheless, cases of infections have become more frequent in people, the causative agents of which are coagulase-negative species *Staphylococcus epidermidis*, *Staphylococcus saprophyticus*, *Staphylococcus haemolyticus*, and *Staphylococcus hominis* [5]. The most common and studied of the coagulase-negative staphylococci is *S. epidermidis*, which is normally a commensal of the skin and mucous surfaces. However, the ability of *S. epidermidis* to form biofilms makes it a serious problem in surgery associated with implanted structures [6].

Notably, *S. epidermidis* and other coagulase-negative staphylococci are an important reservoir of antibiotic resistance genes. Diseases caused by methicillin-resistant staphylococci (MRS) that are resistant to beta-lactams are particularly difficult to treat, especially when methicillin resistance is associated with reduced sensitivity to antibiotics from other classes. For many years, coagulase-negative MRS strains were considered exclusively as hospital pathogens; however, the situation has changed for the worse as these pathogens are increasingly causing community-acquired infections [7,8]. It has been shown that staphylococci with multiple drug resistances (MDR) can be present in wastewater and in other places associated with human activity [9,10].

Most studies of staphylococci in Russia focused on the monitoring of antibiotic resistant isolates in hospitals and were often limited to characterizing only *S. aureus* [11,12,13,14,15]. Only a few studies of environmental, veterinary, and community-associated staphylococci in Russia have been published [16,17]. Data on genomes, virulence factors (VF), and antibiotic resistance genes (ARG) of staphylococci found in Russia are still limited, especially in Siberian Russia, which is located between the Far Eastern and European regions. A few *S. aureus* complete genome studies have been published [17,18,19]. No data regarding the genome characteristics of coagulase-negative staphylococci from Russia have been reported.

In this study, staphylococci found in humans and pets in Novosibirsk, Siberian Russia, were characterized in terms of antibiotic resistance and respective ARGs. In addition, five MDR *S. haemolyticus* and ten *S. epidermidis* isolates were examined for the ability to form biofilms. Their complete genomes were sequenced and analyzed, focusing on the genes responsible for pathogenicity factors and biofilm formation.

## 2. Materials and Methods

### 2.1. Bacterial Strain Isolation and Identification

Clinical specimens and pure cultures were obtained from a number of Novosibirsk hospitals and medical centers, including Railway Clinical Hospital, Department of Surgery of Purulent Wounds; Novosibirsk Research Institute of Traumatology and Orthopedics, Department of Endoscopic Joint Surgery; Scientific Institute of Clinical and Experimental Lymphology, Department of Diabetic Foot Therapy; Center of New Medical Technologies, Department of Gynecology; City Infectious Diseases Clinical Hospital No. 1, Department of Gastroenterology; and the Federal Center of Neurosurgery. The specimens included biopsy material, wound exudate, synovial liquid, cerebrospinal fluid, swabs, urine and fecal samples, skin and mucosal scrapings, sputum, and pure bacterial cultures; the last was obtained from the hospitals. Veterinary isolates were obtained from specimens taken from pets (cats and dogs) in veterinary clinics of Novosibirsk. The origin and amount of clinical and veterinary isolates are listed in Table 1. Ten-fold dilutions of specimens were prepared and the resulting cell suspensions were plated on mannitol salt agar (OXOID, Basingstoke, UK). Cells were grown at 35 °C in an aerobic atmosphere for 18–24 h, and individual colonies were passaged three times under the same conditions. Pure bacterial cultures were obtained, and bacteria of the genus *Staphylococcus* were determined by cultural and morphological characteristics. In each of the specimens, from one to three isolates were obtained, differing in colony morphology, growth rate, and biochemical properties. Pure cultures of staphylococci were deposited in the Collection of Extremophilic Microorganisms and Type Cultures of the Institute of Chemical Biology and Fundamental Medicine Siberian Branch of Russian Academy of Science (CEMTC ICBFM SB RAS).

Strains, conserved at −80 °C in a lysogeny broth medium (LB) containing 25% glycerol, were subcultured in LB medium and on LB agar plates. All cultures were grown at 37 °C for 48 h, and the colonies grown were used for PCR amplification of the 16S rRNA gene. Subsequent identification of the strains was performed by sequencing the 1308-bp PCR fragment of the 16S rRNA gene, as described previously [20]. All PCR amplicons were gel purified (0.6% SeaKem^®^ GTG-agarose, Lonza, ME, USA) and sequenced (BigDye™ Terminator v.3.1 Cycle Sequencing Kit and ABI 3500 Genetic Analyzer, Applied Biosystems, Foster City, CA, USA). The obtained nucleotide sequences of 16S rRNA genes were compared with the corresponding nucleotide sequences extracted from the NCBI GenBank database (http://www.ncbi.nlm.nih.gov, accessed on 1 April 2021). The closest reference sequences of the genus *Staphylococcus* were used with a sequence similarity level of at least 98%, and species names were determined according to a recently published updated classification [21]. Additional identification was done for a number of isolates using GEN III OmniLog Plus ID System (Biolog, Inc., Hayward, CA, USA).

All patient data were collected anonymously. Isolates were considered as hospital specimens if they were obtained from patients who had been in the clinic for at least two weeks and were treated with antibiotics during that time. The isolates were considered as outpatient if they were obtained from patients who were not hospitalized or from patients who had been hospitalized only a few hours. The study was approved by a local ethics Committee of the Center for New Medical Technologies in Novosibirsk; protocol #2, 12 February 2014.

### 2.2. Susceptibility Testing

Susceptibility to five antibiotics was determined using a disk diffusion test according to the guidelines of EUCAST 10.0 (https://eucast.org, accessed on 10 September 2020). Cefoxitin (FOX, 30 µg), amikacin (AMK, 30 µg), gentamicin (CN, 10 µg), erythromycin (E, 15 µg), and clindamycin (DA, 2 µg) were examined. Disks with antibiotics (OXOID) were applied to the lawns of the investigated cultures on Mueller–Hinton agar (OXOID). Cefoxitin was used as a marker for the detection of methicillin resistance and strain *S. aureus* ATCC 25923 was used as a control of susceptibility.

### 2.3. Antibiotic Resistance Genes Detection

Bacterial DNA was extracted from bacterial cultures grown at 35 °C overnight in 2 mL of brain heart infusion broth (BHI Broth, BioMerieux, Marcy-l’Étoile, France) using a DNA extraction kit (Biolabmix, Novosibirsk, Russia) according to the manufacturer’s instructions. Subsequently, all strains were tested for the presence of genes that contribute to the resistance to various classes of antibiotics: *mecA* and *blaZ* genes (beta-lactam and penicillin resistance), *aac(6′)-Ie-aph(2″)-Ia, ant(4′)-Ia*, and *aph(3′)-IIIa* genes (aminoglycoside resistance), and *erm A, erm C*, and *msrA* genes (macrolide and lincosamide resistance) using a set of primers (Table S1).

### 2.4. Study of Biofilm Formation

The study of staphylococcus biofilm formation with the crystal violet staining method was done as described previously [22]. Briefly, an overnight growth culture in tryptic soy broth (TSB) medium (Merch, Darmstadt, Germany) was adjusted to a final OD_600_ of 1.00 ± 0.05 by adding sterile TSB. This OD-adjusted suspension was then diluted 100-fold with TSB containing 1% glucose to obtain the initial bacterial suspension. The wells of a 96-well microplate (TPP, Trasadingen, Switzerland) were inoculated with 200 μL of the suspension and incubated at 35 °C for 24 h. A control well was inoculated with a sterile TSB medium containing 1% of glucose. Each experiment was done in triplicate in three technical repeats. The medium was removed and the wells were washed three times with sterile saline solution. The wells were air dried for 45 min, and the adherent cells were stained with a 0.1% water solution of crystal violet at room temperature for 30 min. Next, the excess crystal violet was removed and the wells were washed five times with 300 μL sterile saline solution. The dye was dissolved with 150 μL of 96% ethanol and the absorbance of each well was read at 595 nm in a microplate reader (Uniplan, Moscow, Russia). The results were processed and interpreted as described previously [22]. In brief, the average OD values (OD_av_) were calculated for all tested strains and for a negative control (non-inoculated medium). The cutoff value (OD_c_) was defined as three standard deviations above the mean OD of the negative control (OD_c_ = OD_av-neg_ + 3 × SD). The strains were divided into three categories, based on the calculated OD_c_: OD_c_ < OD_av_ ≤ 2 × OD_c_ was defined as a weak biofilm producer; 2 × OD_c_ < OD_av_ ≤ 4 × OD_c_ was defined as a moderate biofilm producer; 4OD_c_ ≤ OD_av_ was defined as a strong biofilm producer.

### 2.5. Complete Genome Sequencing and Analysis

Bacterial DNA was extracted from bacterial cultures grown at 35 °C overnight in 10 mL of brain heart infusion broth (BioMerieux, Marcy-l’Étoile, France) using a DNA extraction kit (Biolabmix, Novosibirsk, Russia) according to the manufacturer’s instructions.

The extracted DNA was further used for a paired-end library construction, which was performed using a NEB Next DNA Ultra library prep kit (New England Biolabs, Ipswich, MA, USA). Sequencing was carried out using a MiSeq Benchtop Sequencer (Illumina, Inc., San Diego, CA, USA) and a MiSeq Reagent Kit 2 × 250 v. 2.0 (Illumina, San Diego, CA, USA). The obtained sequences were assembled de novo using the SPAdes genome assembler v.3.15.2 (http://cab.spbu.ru/software/spades, accessed on 1 August 2021). Annotation was performed using Rapid Annotation Subsystem Technology (RAST) v.2.0 (https://rast.nmpdr.org, accessed on 2 September 2021). The genomes and their RAST annotations are located in Supplementary Data S1 and S2, respectively. The obtained sequences were analyzed for the presence of antibiotic resistance genes (ARG) and virulence factors (VF) using a Resistance Genes Identifier (https://card.mcmaster.ca/analyze/rgi, accessed on 2 September 2021) and the Virulence Factor Database (http://www.mgc.ac.cn/VFs, (accessed on 3 September 2021), respectively. In addition, the genomes were checked for the presence of ARGs and VFs manually, using RAST annotation and BLAST search. The sequence types for MDR *Staphylococcus* strains were determined using the PubMLST database (https://pubmlst.org, accessed on 15 September 2021), and the clonal complexes were identified using a BURST analysis [23].

All Staphylococcus sequences/genomes are publicly available through NSBI resources under BioProject PRJNA774949. Unassembled sequencing reads have been deposited in the NCBI Short Read Archive (SRA) under accessions SRR16591584-SRR16591598. Whole Genome Shotgun projects have been deposited at DDBJ/ENA/GenBank under accessions JAJFNL000000000, JAJFNM000000000, JAJFNN000000000, JAJFNO000000000, JAJFNP000000000, JAJFNQ000000000, JAJFNR000000000, JAJFNS000000000, JAJFNT000000000, JAJFNU000000000, JAJFNV000000000, JAJFNW000000000, JAJFNX000000000, JAJFNY000000000, and JAJFNZ000000000. Versions described in this paper are JAJFNL010000000, JAJFNM010000000, JAJFNN010000000, JAJFNO010000000, JAJFNP010000000, JAJFNQ010000000, JAJFNR010000000, JAJFNS010000000, JAJFNT010000000, JAJFNU010000000, JAJFNV010000000, JAJFNW010000000, JAJFNX010000000, JAJFNY010000000, and JAJFNZ010000000.

## 3. Results

### 3.1. Staphylococcus Strain Isolation and Identification

In 2014–2020, a collection of 394 strains of staphylococci from humans and pets (dogs and cats) was isolated in Novosibirsk, Russia (Table 1). Nineteen *Staphylococcus* species were revealed (Table 2), and five strains were identified as belonging to the newly established *Mammaliicoccus* genus [21].

**Figure 1:**
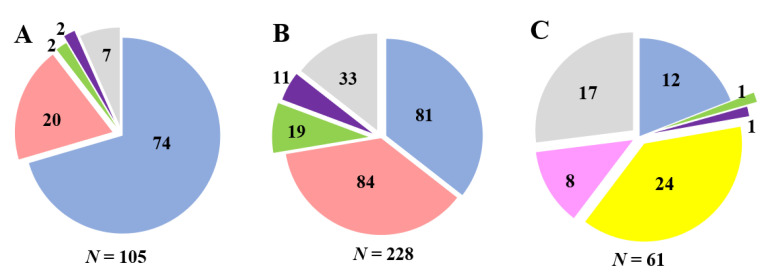
Species distribution of staphylococci isolates from hospitalized patients (**A**), outpatients (**B**), and pets (**C**). Colors: blue—*S. aureus*, light red—*S. epidermidis*, green—*S. haemolyticus,* violet—*S. hominis*, yellow—*S. pseudintermedius*, pink—*S.coagulans*, grey—other staphylococci species. The number of isolates for each species is represented in the circle segments.

*S. aureus*, *S. epidermidis*, *S. haemolyticus*, and *S. hominis* were the major species in clinical samples, whereas *S. pseudintermedius* was the dominant species in veterinary samples. The distribution of coagulase-positive and coagulase-negative staphylococci varied in different panels (Figure 1). Among the strains obtained from hospitalized patients, *S. aureus* strains prevailed (~70%); the share of *S. epidermidis* strains was approximately 19%, and *S. haemolyticus*, *S. warneri*, and other staphylococci accounted for 11%. Outpatient isolates had a different ratio of species: *S. aureus, S. epidermidis*, and *S. haemolyticus* were at approximately 35%, 37%, and 9%, respectively. Veterinary isolates contained ~39%, 20%, and 13% of the *S. pseudintermedius*, *S. aureus*, and *S coagulans* strains, respectively (Figure 1, Table 2). The 16S rRNA gene sequences of all investigated *Staphylococcus* strains (except *S. aureus* strains) were deposited in the NCBI GenBank database. A list of the accession numbers for 16S rRNA gene sequences is given in Table 2.

### 3.2. Antibiotic Resistance and Resistance-Encoding Genes

**Figure 2:**
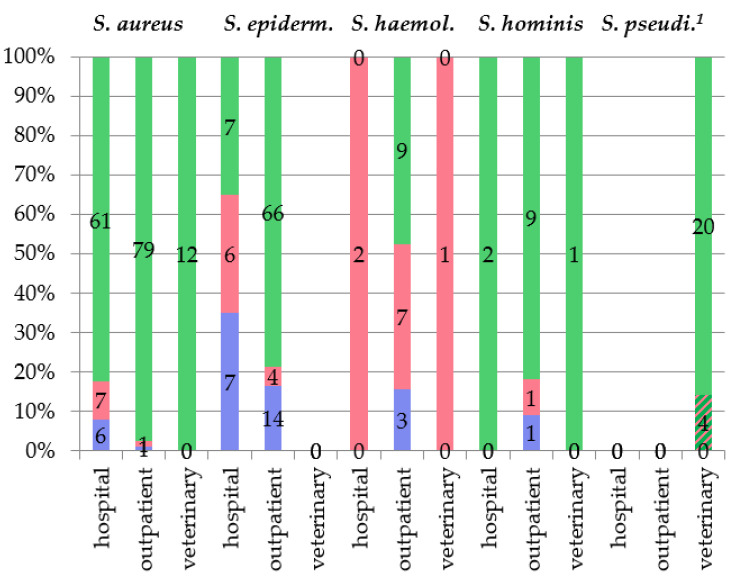
Resistance of predominant *Staphylococcus* species. MSS isolates are marked with green, MDR isolates with rose, and MRS isolates with violet. The number of corresponding isolates is shown on chart columns. ^1^ Chart column marked with diagonal stripes correspond to four MDR *S. pseudintermedius* isolates sensitive to cefoxitin.

All *Staphylococcus* isolates were tested for antibiotic resistance (Figure 2). The majority of them (~85%, 330/389) were methicillin-sensitive (MSS), including 91% of *S. aureus*, 70% of *S. epidermidis*, 85% of *S. hominis*, and all isolates from other species. The exception was *S. haemolyticus* isolates: ~60% (13/22) of them were MRS.

Nevertheless, MRS and MDR isolates resistant to three or more classes of antibiotics were found in all predominant species (Figure 2). All identified MDR isolates were methicillin-resistant, with the exception of *S. pseudintermedius* MDR isolates, which were sensitive to cefoxitin, but resistant to the tested non-beta-lactam drugs. Notably, five of seven *S. aureus* MDR hospital isolates were obtained from purulent diabetic ulcers and six *S. epidermidis* MDR hospital isolates were associated with prosthetic joint and postoperative wound infections. Isolates from the other studied *Staphylococcus* species (*N* = 63) were sensitive to most of the tested antibiotics; however, 10 of 63 commensal isolates were resistant to erythromycin, including all *S. devriesei* isolates, two *S. simulans* isolates, and one isolate each of *M. lentus*, *S. borealis*, *S. warneri*, and *S. pasteuri*. The *S. pasteuri* isolate was also resistant to gentamicin.

In addition, all isolates were assayed for antibiotic resistance genes (ARGs). The presence of the ARGs among the prevailing *Staphylococcus* species is shown in Table 3. The *mecA* gene was identified in all MRS isolates (Table 3); approximately half of all *S. aureus* and half of outpatient *S. epidermidis* isolates contained the *blaZ* gene, encoding penicillin resistance. The majority of aminoglycoside-resistant *Staphylococcus* isolates (59/63) contained a single *aac(6′)-Ie-aph(2″)-Ia* gene (*n* = 34), or its combinations with *aph(3′)-IIIa* (*n* = 14) or *ant(4′)-Ia* (*n* = 11) genes. The erythromycin resistance of *S. aureus* isolates was encoded only by the *ermA/ermC* genes, not *msrA*. In contrast, the outpatient erythromycin-resistant *S. epidermidis* isolates mostly contained *msrA* genes (*n* = 29). A combination of *ermA* and *msrA* was revealed in only one *S. hominis* isolate (Table 3).

In general, there was a clear correlation between the resistance and the presence of the corresponding ARGs. The exception was seven *S. pseudintermedius* erythromycin-resistant isolates and two *S. epidermidis* clindamycin-resistant isolates; in all these isolates the *ermA*, *ermC*, and *msrA* genes were not detected. Probably, other ARGs are responsible for resistance in these isolates. Conversely, genes encoding aminoglycoside resistance were determined in four *S. haemolyticus* and two *S.hominis* isolates; all of them were sensitive to aminoglycosides. The same was observed in erythromycin-sensitive strain of *S. haemolyticus*, in which the *msrA* gene was detected (Table 3). The lack of resistance in these cases is likely due to the mutant variants of the detected gene and/or the lack of its activity.

### 3.3. MDR Isolate Sequence Type Identification

Complete genomes were determined for ten and five MDR isolates of *S. epidermidis* and *S. haemolyticus*, respectively. The sequence types for staphylococcus isolates (Table 4) were determined using the PubMLST database (https://pubmlst.org, accessed on 15 September 2021) and the BURST analysis was used for grouping the isolates into clonal complexes [23]. Eight sequence types were found among ten *S. epidermidis* isolates; six of them were members of the CC5, one (ST23) was determined to be a member of CC23, and two ST 20 isolates were evaluated as singletons. All *S. haemolyticus* isolates were grouped into the clonal complex 3 (Table 4).

### 3.4. Analysis of the Investigated Genomes for the Presence of Antibiotic Resistance Genes

An ARG search was performed using Resistance Gene Identifier (https://card.mcmaster.ca/analyze/rgi, accessed on 2 September 2021). The default selection criteria, which identified genes based on the strict or perfect mode, were used. In addition to those previously identified (Table 3), multiple ARGs have been found in the investigated genomes (Table 5). The main differences between *S. epidermidis* and *S. haemolyticus* isolates were as follows: all *S. epidermidis* genomes contained *norA* (quinolone resistance), *dfrC* (diaminopyrimidine resistance), and *fosB* (fosfomycin resistance), and nine of ten genomes possessed an aminocoumarin-resistant variant of *gyrB*. None of these genes were revealed in *S. haemolyticus* genomes. On the contrary, *msrA* and *mphC* (macrolide resistance) were found in all *S. haemolyticus* genomes but not in *S. epidermidis* genomes. Both the *S. haemolyticus* and *S. epidermidis* genomes had one to three different genes encoding aminoglycoside-modifying enzymes, and the *aac(6′)-Ie-aph(2″)-Ia* gene was identified in all isolates. *FusC* (fusidic acid resistance) and *cat8* (chloramphenicol resistance) were rare and found only in *S. epidermidis* CEMTC 3750 and in *S. haemolyticus* CEMTC1553 isolates (Table 5). Genes encoding vancomycin resistance were not identified; the cluster of bacitracin resistance genes *bce* was found in all studied genomes.

In addition, a number of genes encoding different MDR pumps mediating the efflux were found in the genomes. Among them, genes responsible for biocide resistance were identified, including the gene encoding acriflavin resistance protein (found in all genomes) and *qacA* encoding antiseptic resistance protein (identified in eight *S. epidermidis* and four *S. haemolyticus* genomes, except the 3107, 3117, and 2688 isolates).

### 3.5. Virulence Factor Identification

A number of VFs were identified in the investigated genomes, including genes encoding toxins, exoenzymes, and immune evasion factors (Table 6). All investigated *Staphylococcus* genomes contained the genes *lip* and *nuc*, encoding lipase and thermonuclease, respectively. In addition, two hemolysin-encoding genes (hemolysin III and hemolysin, containing the CBS domain) were found in all studied genomes. The *pgs ABCDE* cluster, responsible for the synthesis of the surface-attached poly-gamma-glutamate (PGA), was also identified in all *S. epidermidis* and *S. haemolyticus* genomes (Table 6). The PGA capsule is produced by a number of coagulase-negative staphylococci and is absent in *S. aureus*. It is a key factor of pathogen survival during infection and efficiently shelters it from components of innate host defense, including antimicrobial peptides and neutrophil phagocytosis [24,25]. Only *S. epidermidis* genomes contained the gene *hlb*, encoding β-hemolysin, and a set of the genes *sspA*, *sspB*, and *geh*, encoding exoenzymes.

The number of virulence factors in the studied *S. epidermidis* genomes was lower compared to the known pathogenic *S. aureus* genomes. This is typical for coagulase-negative staphylococci, which are usually not as pathogenic as *S. aureus* isolates [5]. Notably, the number of VFs identified in the genomes of *S. haemolyticus* was lower than in the genomes of studied *S. epidermidis*, which may partially be due to insufficient information about the virulence factors of *S. haemolyticus* [5].

At the same time, a capsular of investigated *S. haemolyticus* isolates probably has a more complex structure compared to *S. epidermidis*, as additional genes encoding enzymes of capsular synthesis were revealed in the genomes of *S. haemolyticus* strains CEMTC 2119 and CEMTC 3413 (Table 6). These genes possess more than 50% similarity with the closely related *cap5* and *cap8* gene clusters of *S. aureus*, which are responsible for CP5 and CP8 (the two main CP serotypes usual for clinical *S. aureus* strains) [26,27].

### 3.6. In Vitro Biofilm Formation by MDR S. epidermidis and S. haemolyticus Isolates and Factors Responsible for Adhesion and Biofilm Formation

**Figure 3:**
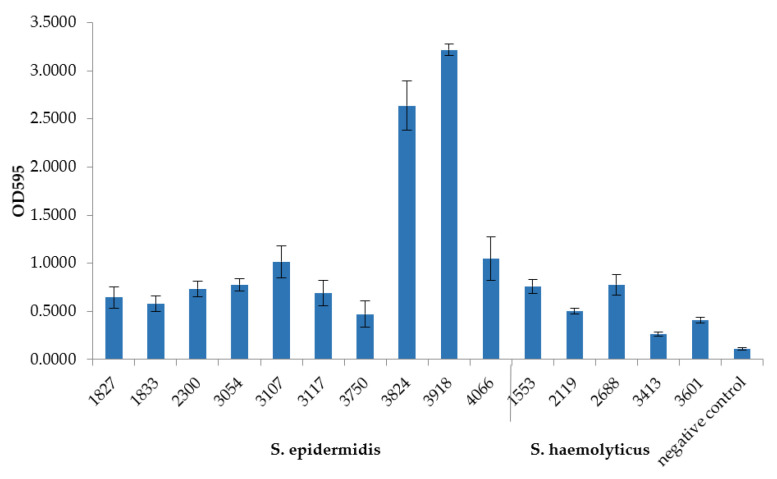
Biofilm formation by MDR *S. epidermidis* and *S. haemolyticus* isolates.

The studied isolates have demonstrated a different ability to form biofilms (Figure 3). All isolates were divided into three categories (weak, moderate, and strong biofilm producers), as described previously [22]. Based on the cutoff value (OD_c_), set as three standard deviations above the mean OD of negative control and calculated as 0.15, *S. haemolyticus* CEMTC 3413 was classified as a weak biofilm producer (0.15 < OD_av_ ≤ 0.3), *S. epidermidis* CEMTC 1833, CEMTC 3750, and *S. haemolyticus* CEMTC 2119, CEMTC 3601 were defined as moderate biofilm producers (0.3 < OD_av_ ≤ 0.6), and the remaining isolates were defined as strong biofilm producers (0.6 ≤ OD_av_). The *S. epidermidis* CEMTC 3824 and CEMTC 3918 isolates showed the highest biofilm formation.

Two distinct mechanisms of biofilm formation have been identified previously. One involves a number of cell-wall-associated proteins (CWA), responsible for adhesion to the host cells or abiotic surfaces and subsequent biofilm production, and the other requires the synthesis and secretion of a polysaccharide intercellular adhesin (PIA) [28,29]. The genome analysis revealed a number of genes that promote biofilm formation (Table 7). All *S. epidermidis* and *S. haemolyticus* genomes contained *atlE* (autolysin), *ebpS* (elastin binding protein), and *sas* family genes, all of them encoding CWA proteins responsible for adhesion. A number of *sdr* family genes, encoding Ser-Asp-rich fibrinogen-binding proteins, were found in all *S. epidermidis* genomes; however, two of five *S. haemolyticus* genomes contained a single *sdrC* gene. The *aap* gene (which encodes an accumulation-associated protein, Aap) was found in the genomes of all *S. epidermidis* strains capable of strong biofilm production, except strain 1827.

No clear correlation between the presence of *ica*-operon and the ability to form biofilms was found (Table 7, Figure 3). Six *S. epidermidis* genomes contained *ica*-operon, associated with a high level of biofilm formation. Most of them, except strain 1833, were strong biofilm producers. Notably, *S. epidermidis* strains 3824 and 3918, which differed considerably from the others in terms of their ability to produce biofilms (Figure 3), had a combination of *ica*-operon, *aap*, *pls*, and four *sdr*-genes. At the same time, genes encoding phenol-soluble modulins (PSMs) were absent from both strains (Table 7). PSMs disrupt non-covalent bonds between cells inside the biofilm, promoting channel formation and cell release from the mature biofilm; mutant *S. epidermidis* strains, lacking PSM, were unable to form channels and produced more substantial biofilms [31,32,33].

In general, the investigated *S. epidermidis* strains were strong or moderate biofilm producers and their genomes contained multiple genes responsible for this function. At the same time, only two of five *S. haemolyticus* strains were capable of intense biofilm formation, and the well-known genes responsible for the formation of biofilms were poorly represented in their genomes. Presumably, their genomes contain some unidentified genes responsible for biofilm formation. Notably, both *S. haemolyticus* strains CEMTC 3413 and CEMTC 2119, possessing weak or moderate ability for biofilm formation, contained a putative capsule synthesis operon (Table 7). This may be one of the reasons for the weak biofilm formation—only non-encapsulated cells are able to adhere to the extracellular matrix or to endothelial cells [34].

## 4. Discussion

The widespread, sometimes unjustified use of antibiotics in recent decades has led to a significant increase in the proportion of antibiotic-resistant and MDR strains among nosocomial agents [35,36,37,38]. In this study, a wide spectrum of staphylococci species found in hospitalized patients and outpatients in Novosibirsk (with more than 1.5 million inhabitants) were investigated for their antibiotic resistance. In addition, staphylococci isolated from pets in close contact with humans were involved in the study, because such staphylococci can be a reservoir of pathogenic MRS and MDR strains [39,40].

Only two coagulase-positive species were identified: *S. aureus* (in humans and pets) and *S. pseudintermedius* (only in pets); such staphylococci dominated in samples from hospitalized patients and pets (~70% and ~60%, respectively). Seventeen coagulase-negative staphylococci were revealed, with the most variety in outpatients (15 species), where coagulase-negative staphylococci were in the majority (~75%). Two coagulase-negative species, *S. coagulans* and *S. felis*, were found only in veterinary samples.

Our investigation showed that the majority of isolates of all found species, with the exception of *S. haemolyticus* and hospital *S. epidermidis* isolates, were sensitive to most of the antibiotics tested and the number of MRS and MDR strains was not high. Most *S. haemolyticus* isolates were MRS, which corresponded to the known data on a high level of methicillin resistance in *S. haemolyticus* [5]. The animal isolates had the lowest antibiotic resistance to tested antibiotics, and only one veterinary *S. haemolyticus* MRS and four *S. pseudintermedius* MDR isolates (all methicillin-sensitive) were found among 61 isolates from domestic animals. Nevertheless, MRS and/or MDR isolates were found in all prevailed species, including hospital, outpatient, and veterinary isolates.

The data on MDR and MRS coagulase-negative staphylococci in different countries and regions are heterogeneous and depend on the country, region, type of hospital, etc. It is also known that hospital, community-associated, and environmental coagulase-negative isolates differ significantly in their antibiotic resistance, with the highest level of resistance a characteristic of hospital isolates. Unfortunately, the situation is changing for the worse in recent years. For instance, 17% of community-associated isolates, 30% of healthcare personnel isolates, and ~87% of hospital *S. epidermidis* isolates were MRS in 2013 in Shanghai, China [41]. In 2018, a higher percentage of MRSE isolates (76.5%) was recovered from healthcare personnel in two public hospitals in Tianjin, China [42]. Another study revealed that 20.5% of environmental CoNS staphylococci detected in the environment of a university in Thailand were MRS and 61.0% of these MRS isolates were MDR [43].

The beta-lactam resistance was mediated by the *mecA* gene, which was found in all MRS isolates. Aminoglycoside-modifying enzymes were represented in all species and encoded mainly by the *aac(6′)-Ie-aph(2″)-Ia* gene and its combination with *aph(3′)-IIIa* or *ant(4′)-Ia* genes. At the same time, the macrolide resistance genes distribution was variable: only *ermA/ermC* genes were found in *S. aureus* isolates; in contrast, macrolide-resistant *S. epidermidis*, *S. haemolyticus*, and *S. hominis* isolates were mostly found to contain the *msrA* gene. Perhaps this indicates separate ways of the resistance genes transfer between different species of coagulase-negative staphylococci. None of the macrolide-resistant veterinary *S. pseudintermedius* isolates contained *ermA/ermC* or *msrA* genes. Other mechanisms (efflux pump or antibiotic-modifying enzymes) likely mediated macrolide resistance in these isolates.

Complete genome sequencing and analysis, which was performed for ten MDR *S. epidermidis* and five MDR *S. haemolyticus* isolates, identified other ARGs and genes associated with biofilm formation and virulence. The species-specific distribution of a number of ARGs was revealed in the genomes; *norA*, *dfrC,* and *fosB* genes were found in all *S. epidermidis* genomes, whereas *mphC* and *msrA* were identified in all *S. haemolyticus* ones.

The distribution of the macrolide-resistance-encoding genes *msrA* and *ermA/ermC*, which were found in complete genomes, was in contrast to the usual distribution of these genes among *S. epidermidis* isolates. Notably, almost all macrolide-resistant *S. epidermidis* isolates for which genome sequences were determined in this study were isolated from hospitalized patients; possibly, *ermA/ermC* genes were transmitted to *S. epidermidis* isolates from the nosocomial strains of *S. aureus.*

Six *S. epidermidis* MDR hospital isolates were associated with prosthetic joint and post-operative wound infections. The last association may be mediated by one of the major factors of *S. epidermidis* pathogenicity, their biofilm formation ability [5,6]. Indeed, four of the six *S. epidermidis* MDR hospital isolates were strong biofilm producers and the remaining two isolates demonstrated moderate biofilm-forming activity. In addition, all these strains contained the antiseptic resistance gene *qacA* and multiple ARGs. All of these factors were found to correlate with poor patient outcome, as was shown previously [6].

In addition, the MLST profiling revealed the genetic diversity of isolates: eight different ST were found among ten *S. epidermidis* isolates. The observed genetic diversity is in accordance with previously published data [44,45]. The majority of identified *S. epidermidis* sequence types belonged to clonal complex CC5, which has been previously reported as one of the leading causative agents for bloodstream and prosthetic joint infections [45,46,47]. A recent study [48] has shown that ST5 isolates can be detected as matched commensal/invasive pairings in the same human organism, unlike ST2, which is probably purely a hospital pathogen. Presumably, ST5 isolates are able to evolve in one organism from commensal to infectious variants.

A number of *S. epidermidis* isolates have been previously reported in Russia (https://pubmlst.org, accessed on 15 September 2021). Among the sequence types identified in this study, the ST5, ST20, and ST23 isolates were found in 2008–2009 in Moscow and Nizhny Novgorod, European Russia, whereas ST786 was obtained from a hospitalized patient in 2018 in Moscow [49]. Others, namely ST17, ST152, and ST210, were found for the first time in Russia. All eight STs were revealed previously as both colonizing and infectious agents. Isolate ST210 was reported at first to be associated with samples taken from healthy people [41]; however, a number of reports of ST210 isolates causing infection were subsequently published [50,51]. The ST17, ST20, and ST23 isolates were previously identified as both colonizing and infectious agents [44]. A set of ST152 isolates were previously revealed as human-colonizing staphylococci (isolated in the USA) and as mastitis-associated agents in cows (isolated in Greece), whereas ST786 was identified in China as a community-associated isolate (https://pubmlst.org, accessed on 15 September 2021).

*S. haemolyticus* isolates were identified as belonging to four different ST; all of them were grouped into CC3. STs of *S. haemolyticus* isolates from Russia have not been previously reported (https://pubmlst.org, accessed on 15 September 2021).

In conclusion, the majority of isolates of 19 staphylococci species found in Novosibirsk in 2014–2020 were sensitive to most of the tested antibiotics. The exception was *S.* *haemolyticus* and hospital *S. epidermidis* isolates. Nevertheless, MRS and/or MDR isolates were found in all prevailed (*S. aureus*, *S. epidermidis*, *S. haemolyticus*, *S. hominis*, and *S. pseudintermedius*) species. ARGs (*mecA*, *aac(6′)-Ie-aph(2″)-Ia*, *aph(3′)-IIIa*, *ant(4′)-Ia*, *ermA/ermC*, and *msrA*) were identified in staphylococcus isolates, and a clear correlation with the corresponding resistance was revealed. Complete genomes were sequenced and analyzed for ten MDR *S. epidermidis* and five MDR *S. haemolyticus* isolates. Antibiotic resistance genes *mphC*, *qacA/qacB*, *norA*, *dfrC/dfrG*, *lnuA*, *BseSR*, *fosB,* and multiple genes responsible for virulence and pathogenicity were found. All investigated MDR *S. epidermidis* and four of five *S. haemolyticus* strains were moderate or strong biofilm producers, whereas multiple genes responsible for this function were identified mostly in *S. epidermidis* genomes and were less represented in *S. haemolyticus* genomes.

## Figures and Tables

**Table 1:** The origin of *Staphylococcus* isolates.

Panels	Origin of Strains	Number of Isolates
Strains isolated from hospitalized patients (*n* = 105)	Prosthetic joint and post-operative wound infections	56
	Purulent diabetic ulcers	43
	Decubitus ulcers	3
	Sputum from a patient with pneumonia	2
	Cerebrospinal fluid	1
Strains from outpatients (*n* = 228)	Respiratory tract infections	83
	Skin and mucosal infections	59
	Urinary tract infections	21
	Bacterial vaginosis	25
	Fecal samples from patients with diarrhea	36
	Purulent diabetic ulcers	4
Strains from pets (39 from dogs and 22 from cats) (*n* = 61)	Skin and mucosal infections	57
	Fecal samples from animals with diarrhea	4

**Table 2:** Species identification of clinical and veterinary staphylococci isolates.

No	Species (Number of Isolates)	Isolation Source (Number of Isolates)	GenBank Identifier for 16S rRNA Gene
1	*Staphylococcus aureus* (167)	Hospital (74)	No ID
		Outpatient (81)	No ID
		Veterinary (12)	No ID
2	*Staphylococcus auricularis* (4)	Outpatient (4)	MZ014399–MZ014402
3	*Staphylococcus borealis* (2)	Outpatient (2)	MZ014403, MZ014404
4	*Staphylococcus capitis* (4)	Outpatient (4)	MZ014405–MZ014407, MZ014411
5	*Staphylococcus caprae* (5)	Hospital (1)	MZ014409
		Outpatient (4)	MZ014408, MZ014410, MZ014412, MZ014413
6	*Staphylococcus carnosus* (2)	Outpatient (2)	MZ014414, MZ014415
7	*Staphylococcus casei* (2)	Outpatient (1)	MZ014416
8	*Staphylococcus coagulans/S. schleiferi* subsp. *coagulans* (8)	Veterinary (8)	MW979964–MW979970
9	*Staphylococcus cohnii* (2)	Outpatient (2)	MZ014417, MZ014418
10	*Staphylococcus devriesei* (4)	Outpatient (4)	MZ014419–MZ014422
11	*Staphylococcus equorum* (3)	Outpatient (1)	MZ014423
		Veterinary (2)	MW979955, MW979971
12	*Staphylococcus epidermidis* (104)	Hospital (20)	MZ027385–MZ027397, MZ040881, MZ040882, MZ041685
		Outpatient (84)	MZ027349–MZ027358, MZ027360–MZ027362, MZ027364–MZ027366, MZ040893–MZ040916, MZ047203–MZ047210, MZ049531–MZ049537,MZ723062
13	*Staphylococcus felis* (5)	Veterinary (5)	MW979972–MW979976
14	*Staphylococcus haemolyticus* (22)	Hospital (2)	MZ027398, MZ027399
		Outpatient (19)	MZ027367–MZ027375, MZ040917–MZ040921, MZ047213–MZ047216, MZ723059, MZ723059
		Veterinary (1)	MW979956
15	*Staphylococcus hominis* (14)	Hospital (2)	MZ014434, MZ014435
		Outpatient (11)	MZ014424–MZ014433
		Veterinary (1)	MW979977
16	*Staphylococcus pasteuri* (1)	Outpatient (1)	MZ047217
17	*Staphylococcus pseudintermedius* (24)	Veterinary (24)	MW965646, MW979953, MW979978–MW979995, MW979997, MW979998
18	*Staphylococcus simulans* (8)	Hospital (1)	MZ014437
		Outpatient (5)	MZ014436, MZ014438–MZ014441
		Veterinary (2)	MW979999, MW980000
19	*Staphylococcus warneri* (8)	Hospital (2)	MZ014445, MZ014447
		Outpatient (5)	MZ014442–MZ014444, MZ014446, MZ014448
		Veterinary (1)	MW979957
20	*Mammaliicoccus sciuri/Staphylococcus sciuri* (3) ^1^	Hospital (1)	MW965541
		Veterinary (2)	MW965545, MW965551
21	*Mammaliicoccus vitulinus/Staphylococcus vitulinus* (1) ^1^	Veterinary (1)	MW965552
22	*Mammaliicoccus lentus/Staphylococcus lentus* (1) ^1^	Veterinary (1)	MW965543

^1^ The species were moved to the newly established *Mammaliicoccus* genus [21], previous species names are separated by slash.

**Table 3:** Antimicrobial resistance profiles and resistance-encoding genes of prevailing *Staphylococcus* isolates.

Antimicrobial	Resistance-Encoding Gene(s) ^1^	Number of Isolates with Resistance Profiles and ARGs That Were Identified in These Isolates		
*S. aureus*				
		Hospital (*n* = 74)	Outpatient (*n* = 81)	Veterinary (*n* = 12)
Cefoxitin		13	2	0
	*mecA*	13	2	0
Penicillin		N.d ^2^	N.d.	N.d.
	*blaZ*	32	38	3
Gentamicin and/or Amikacin		12	2	0
	*aac(6′)-Ie-aph(2″)-Ia*	8	1	0
	*aac(6′)-Ie-aph(2″)-Ia + ant(4′)-Ia*	1	1	0
	*aac(6′)-Ie-aph(2″)-Ia + aph(3′)-IIIa*	3	0	0
Erythromycin		0	5	0
	*ermC*	0	5	0
Erythromycin and Clindamycin		7	5	0
	*ermA*	5	0	0
	*ermC*	2	5	0
*S. epidermidis*				
		Hospital (*n* = 20)	Outpatient (*n* = 84)	Veterinary (*n* = 0)
Cefoxitin		13	18	0
	*mecA*	13	18	0
Penicillin		N.d.	N.d.	N.d.
	*blaZ*	6	39	0
Gentamicin and/or Amikacin		9	19	0
	*aac(6′)-Ie-aph(2″)-Ia*	4	11	0
	*ant(4′)-Ia*	0	3	0
	*aac(6′)-Ie-aph(2″)-Ia + ant(4′)-Ia*	5	2	0
	*aac(6′)-Ie-aph(2″)-Ia + aph(3′)-IIIa*	0	3	0
Erythromycin		6	34	0
	*ermC*	2	5	0
	*msrA*	4	29	0
Clindamycin		1 ^3^	1 ^3^	0
Erythromycin and Clindamycin		4	2	0
	*ermA*	3	0	0
	*ermC*	1	0	0
	*msrA*	0	2	0
*S. haemolyticus*				
		Hospital (*n* = 2)	Outpatient (*n* = 19)	Veterinary (*n* = 1)
Cefoxitin		2	10	1
	*mecA*	2	10	1
Penicillin		N.d.	N.d.	N.d.
	*blaZ*	1	3	0
Gentamicin and/or Amikacin		2	10	1
	*aac(6′)-Ie-aph(2″)-Ia*	1	7	1
	*ant(4′)-Ia*	0	4 ^4^	0
	*aac(6′)-Ie-aph(2″)-Ia + aph(3′)-IIIa*	1	3	0
Erythromycin		2	13	0
	*ermC*	0	3	0
	*msrA*	2	10	1 ^4^
*S. hominis*				
		Hospital (*n* = 2)	Outpatient (*n* = 19)	Veterinary (*n* = 1)
Cefoxitin		0	2	0
	*mecA*	0	2	0
Penicillin		N.d.	N.d.	N.d.
	*blaZ*	1	3	0
Gentamicin and/or Amikacin		0	2	0
	*ant(4′)-Ia*	0	1 ^4^	0
	*aph(3′)-IIIa*	0	0	1 ^4^
	*aac(6′)-Ie-aph(2* *″* *)-Ia + ant(4′)-Ia*	0	2	0
Erythromycin		0	5	0
	*msrA*	0	4	0
	*ermA + msrA*	0	1	0
*S. pseudintermedius*				
		Hospital (*n* = 0)	Outpatient (*n* = 0)	Veterinary (*n* = 24)
Penicillin		N.d.	N.d.	N.d.
	*blaZ*	0	0	4
Gentamicin and/or Amikacin		0	0	6
	*aac(6′)-Ie-aph(2″)-Ia*	0	0	1
	*aph(3′)-IIIa*	0	0	1
	*aac(6′)-Ie-aph(2″)-Ia + aph(3′)-IIIa*	0	0	4
Erythromycin and Clindamycin		0	0	7 ^3^

^1^*mecA*, *aac(6′)-Ie-aph(2″)-Ia*, *aph(3′)-IIIa, ant(4′)-Ia*, *ermA/ermC*, and *msrA* genes were checked in all isolates; ^2^ not determined; ^3^ isolates had clindamycin or erythromycin resistant profiles, but investigated genes were not found; ^4^ isolates contained genes, encoding aminoglycoside resistance, but had no aminoglycoside-resistant phenotype.

**Table 4:** Source of isolation, resistance characteristics, and sequence types of MDR *S. epidermidis* and MDR *S. haemolyticus* isolates.

No	Species	CEMTC No of Isolate/16S rRNA GenBank ID	Source of Isolation	Resistance	Sequence Type	Clonal Complex
1	*S. epidermidis*	1827/MZ027386	Hospital (swab from purulent diabetic ulcer)	FOX, AK, CN, E, DA	ST 23	CC23
2	*S. epidermidis*	1833/MZ027390	Hospital (pure culture, post-operative wound infection)	FOX, AK, CN, E, DA	ST 23	CC23
3	*S. epidermidis*	2300/MZ027350	Outpatient (nasal swab, rhinitis)	FOX, AK, CN	ST 20	S ^1^
4	*S. epidermidis*	3054/MZ027389	Hospital (pure culture, post-operative wound infection)	FOX, CN, E, DA	ST 5	CC5
5	*S. epidermidis*	3107/MZ027352	Outpatient (nasal swab, rhinitis)	FOX, CN, E	ST 152	CC5
6	*S. epidermidis*	3117/MZ027364	Outpatient (faeces, diarrhea)	FOX, CN, E	ST 152	CC5
7	*S. epidermidis*	3750/MZ027394	Hospital (biopsy material, prosthetic joint infection)	FOX, AK, CN	ST 210	CC5
8	*S. epidermidis*	3824/MZ027395	Hospital (biopsy material, prosthetic joint infection)	FOX, AK, CN, E	ST 786	CC5
9	*S. epidermidis*	3918/MZ027396	Hospital (pure culture, post-operative wound infection)	FOX, AK, CN, E	ST 20	S ^1^
10	*S. epidermidis*	4066/MZ027397	Hospital (pure culture, post-operative wound infection)	FOX, AK, CN, E, DA	ST 17	CC5
11	*S. haemolyticus*	1553/MZ027371	Outpatient (urine sample, pyelonephritis)	FOX, AK, CN, E	ST 1	CC3
12	*S. haemolyticus*	2119/MZ027368	Outpatient (purulent diabetic ulcer)	FOX, CN, E	ST 3	CC3
13	*S. haemolyticus*	2688/MZ723059	Outpatient (faeces, diarrhea)	FOX, CN, E	ST 3	CC3
14	*S. haemolyticus*	3413/MZ027399	Hospital (sputum, ventilator-associated pneumonia)	FOX, AK, CN, E	ST 42	CC3
15	*S. haemolyticus*	3601/MZ027370	Outpatient (skin infection, skin scraping)	FOX, AK, CN, E	ST 8	CC3

^1^ Singleton.

**Table 5:** Antibiotic resistance genes revealed in the genomes of MDR *S. epidermidis* and *S. haemolyticus* isolates.

Antibiotic Classes	ARG	*S. epidermidis* Isolates ^1^										*S. haemolyticus* Isolates ^1^				
		1827	1833	2300	3054	3107	3117	3750	3824	3918	4066	1553	2119	2688	3413	3601
Penicillins	*blaZ*		✓ ^2^	✓			✓	✓	✓	✓	✓	✓	✓	✓	✓	
Cephalosporins	*mecA*	✓	✓	✓	✓	✓	✓	✓	✓	✓	✓	✓	✓	✓	✓	✓
	*mecI*			✓												
	*mecR1*	✓		✓	✓					✓	✓					
Macrolides and Lincosamides	*ermA*	✓	✓								✓					
	*ermC*				✓	✓	✓	✓		✓						
	*msrA*											✓	✓	✓	✓	✓
	*mphC*											✓	✓	✓	✓	✓
Aminoglycosides	*aph(3′)-IIIa*											✓			✓	✓
	*ant(4′)-Ia*	✓	✓	✓				✓	✓	✓						
	*aac(6’)-Ie-aph(2″)-Ia*	✓	✓	✓	✓	✓	✓	✓	✓	✓	✓	✓	✓	✓	✓	✓
	*aad(6)*											✓			✓	
	*ant(9)*	✓	✓								✓					
Quinolones	*norA*	✓	✓	✓	✓	✓	✓	✓	✓	✓	✓					
	*qacA*			✓	✓			✓	✓	✓		✓	✓		✓	✓
	*qacB*	✓	✓								✓					
Chlorampheni- col	*catA8*											✓				
	*catA7*	✓	✓										✓	✓	✓	✓
Fusidic acid	*fusC*							✓								
Diaminopyrimi- dines	*dfrC*	✓	✓	✓	✓	✓	✓	✓	✓	✓	✓					
	*dfrG*					✓	✓	✓					✓		✓	
Aminocoumarins	Aminocoumarin resistant *gyrB*	✓	✓	✓	✓	✓	✓	✓	✓		✓					
Lincosamides	*lnuA*			✓				✓	✓	✓			✓	✓		✓
Tetracyclines	*tet(K)*			✓				✓								✓
	*tet(45)*									✓						
Fosfomycin	*fosB*	✓	✓	✓	✓	✓	✓	✓	✓	✓	✓					
Bacitracin	*BceSR*	✓	✓	✓	✓	✓	✓	✓	✓	✓	✓	✓	✓	✓	✓	✓

^1^*S. epidermidis* isolates are marked with blue, *S. haemolyticus* isolates are marked with green; ^2^ the “tick” symbol means that corresponding antibiotic resistance gene was revealed in the isolate.

**Table 6:** Virulence factors identified in the genomes of *S. epidermidis* and *S. haemolyticus* isolates.

Species and No in CEMTC	Exoenzymes				Toxins			Immune Evasion
	Cysteine Protease	Serine V8 Protease	Lipases	Thermonuclease	Hemo- lysin III	Hemolysin, Containing CBS Domain	β-Hemolysin	PGA, Capsule
*S. epidermidis* 1827	*sspB*	*sspA*	*geh, lip*	*nuc*	+ ^1^	+ ^1^	*hlb*	*capA, pgsABCDE* ^2^
*S. epidermidis* 1833	*sspB*	*sspA*	*geh, lip*	*nuc*	+	+	*hlb*	*capA, pgsABCDE*
*S. epidermidis* 2300	*sspB*	*sspA*	*geh, lip*	*nuc*	+	+	*hlb*	*capA, pgsABCDE*
*S. epidermidis* 3054	*sspB*	*sspA*	*geh, lip*	*nuc*	+	+	*hlb*	*capA, pgsABCDE*
*S. epidermidis* 3107	*sspB*	*sspA*	*geh, lip*	*nuc*	+	+	*hlb*	*capA, pgsABCDE*
*S. epidermidis* 3117	*sspB*	*sspA*	*geh, lip*	*nuc*	+	+	*hlb*	*capA, pgsABCDE*
*S. epidermidis* 3750	*sspB*	*sspA*	*geh, lip*	*nuc*	+	+	*hlb*	*capA, pgsABCDE*
*S. epidermidis* 3824	*sspB*	*sspA*	*geh, lip*	*nuc*	+	+	*hlb*	*capA, pgsABCDE*
*S. epidermidis* 3918	*sspB*	*sspA*	*geh, lip*	*nuc*	+	+	*hlb*	*capA, pgsABCDE*
*S. epidermidis* 4066	*sspB*	*sspA*	*geh, lip*	*nuc*	+	+	*hlb*	*capA, pgsABCDE*
*S. haemolyticus* 1553			*lip*	*nuc*	+	+		*capA, pgsABCDE*
*S. haemolyticus* 2119	*sspB*	*sspA*	*geh, lip*	*nuc*	+	+		*capA, pgsABCDE, cap5L, cap5F, cap8C, cap8E, cap8I, cap8H, cap 8M, cap8N* ^2^
*S. haemolyticus* 2688			*lip*	*nuc*	+	+		*capA, pgsABCDE, cap5A*
*S. haemolyticus* 3413			*lip*	*nuc*	+	+		*capA, pgsABCDE, cap5L, cap5F, cap8C, cap8E, cap8I, cap8H, cap 8M, cap8N* ^2^
*S. haemolyticus* 3601			*lip*	*nuc*	+	+		*capA, pgsABCDE, cap5A, cap8C* ^3^

^1^ the “plus” symbol means that gene, encoding the appropriate hemolysin, was revealed in the isolate; ^2^ *pgsABCDE* cluster responsible for synthesis of the surface-attached poly-gamma-glutamate (PGA); ^3^ putative capsule operon, possessing ≥50% identity to *S. aureus cap5* or *cap8* locus.

**Table 7:** Adherence and biofilm formation factors, identified in the genomes of MDR *S. epidermidis* and *S. haemolyticus* isolates.

Species and No in CEMTC	Ability to Form Biofilm	Adherence and Biofilm Formation Genes								
		PIA	PSM	Aap	Pls	Sas-Family Proteins	Autoly- sin	Fibronectin Binding Protein ^1^	Elastin Binding Protein	Sdr-Family Proteins
*S. epidermidis* 1827	strong	*icaADBC, icaR*	*psmβ1, psmβ2*		*pls*	*SasA, SasC, SasF*	*atlE*	*ebh*	*ebp*	*sdrG, sdrH*
*S. epidermidis* 1833	moderate	*icaADBC, icaR*			*pls*	*SasA, SasC, SasF*	*atlE*	*ebh*	*ebp*	*sdrG*
*S. epidermidis* 2300	strong	*icaADBC, icaR*	*psmβ1, psmβ2*	*aap*	*pls*	*SasA, SasC, SasF*	*atlE*	*ebh*	*ebp*	*sdrC, sdrF, sdrH*
*S. epidermidis* 3054	strong		*psmβ1, psmβ2*	*aap*		*SasA, SasC, SasF*	*atlE*	*ebh*	*ebp*	*sdrF, sdrG, sdrH*
*S. epidermidis* 3107	strong		*psmβ1, psmβ2*	*aap*		*SasA, SasC, SasF*	*atlE*	*ebh*	*ebp*	*sdrF, sdrG, sdrH*
*S. epidermidis* 3117	strong		*psmβ1, psmβ2*	*aap*		*SasA, SasC, SasF*	*atlE*	*ebh*	*ebp*	*sdrF, sdrG, sdrH*
*S. epidermidis* 3750	moderate		*psmβ1, psmβ2*		*pls*	*SasA, SasC, SasF*	*atlE*	*ebh*	*ebp*	*sdrE, sdrF, sdrG, sdrH*
*S. epidermidis* 3824	strong	*icaADBC, icaR*		*aap*	*pls*	*SasA, SasC, SasF*	*atlE*		*ebp*	*sdrE, sdrF, sdrG, sdrH*
*S. epidermidis* 3918	strong	*icaADBC, icaR*		*aap*	*pls*	*SasA, SasC, SasF*, *SasG*	*atlE*	*ebh*	*ebp*	*sdrE, sdrF, sdrG, sdrH*
*S. epidermidis* 4066	strong	*icaADBC, icaR*	*psmβ1, psmβ2*	*aap*		*SasA, SasC, SasF*	*atlE*		*ebp*	*sdrF, sdrG, sdrH*
*S. haemolyticus* 1553	strong			*aap*		*SasA, SasC, SasF*, *SasG*	*atlE*		*ebp*	
*S. haemolyticus* 2119	moderate					*SasA, SasC, SasF*, *SasG*	*atlE*		*ebp*	
*S. haemolyticus* 2688	strong					*SasA, SasC, SasF*, *SasG*	*atlE*		*ebp*	
*S. haemolyticus* 3413	weak			*aap*		*SasA, SasC, SasF*, *SasG*	*atlE*		*ebp*	*sdrC*
*S. haemolyticus* 3601	moderate					*SasA, SasC, SasF*, *SasG*	*atlE*		*ebp*	*sdrC*

^1^ Extracellular matrix-binding protein (Embp) homologue [30].

## Data Availability

The data presented in this study are available in supplementary, Data S1: Staphylococcus genome sequences; Data S2: RAST annotation of the genomes.

## Supplementary Materials

The following are available online at https://www.mdpi.com/article/10.3390/microorganisms9122487/s1, Table S1: Primers, specific to antibiotic resistance encoding genes; Data S1: Staphylococcus genome sequences; Data S2: RAST annotation of the genomes.
