# Supplementary material for: Antibiotic Resistance and Pathogenomics of Staphylococci Circulating in Novosibirsk, Russia
Source: Microorganisms. 2021 Nov 30;9(12):2487. doi: 10.3390/microorganisms9122487 (PMC8706439; doi:10.3390/microorganisms9122487)
Supplement: Supplementary file 1 [file microorganisms-09-02487-s001.zip › Table S1.pdf]

Table S1. Primers, specific to antibiotic resistance encoding genes

Gene	Primer	Sequence
blaZ	blaZ	5`-(HEX)-AGTCTTGCCGAAAGCAGCAGGCGTTGAAGT-(BHQ1)-3`
	Fbz	5`-TGGGAGATAAAGTAACAAATCCAGT-3`
	Rbz	5`-GCTTAATTTTCCATTTGCGATAAGT-3`
mecA	Zmec	5`-(HEX)-GCTCCAACATGAAGATGGCTATCGTGTCACAATCGT-(BHQ1)-3`
	Fmec	5`-AGGGACTCGAAAAACTTTACGAT-3`
	Rmec	5`-ATTAATGTATGTGCGATTGTATTGC-3`
ermA	ErmA	5`-(HEX)-TCATCCTAAGCCAAGTGTAGACTCTGT-(BHQ1)-3`
	Rera	5`-TTGATGTCGTTCAAGAACAATCA-3`
	Fera	5`-GCTCAAAAAAGTACCACCACT-3`
ermC	ermC	5`-(HEX)-AGAGGTGTAATTTCGTAACTGCCATTGAAATAGACC-(BHQ1)-3`
	Ferc	5`-GGGCATTTTACCCTTGAATTAGT-3`
	Rerc	5`-ACAAGTTTATTTTCTGTAGTCTTGCA-3`
msrA	msrA	5`-(HEX)-ACCTACACCATTTGCACCTACGAGCGCT-(BHQ1)-3`
	Fmsr	5`-CTGCTAACACAAGTACGATTCC-3`
	Rmsr	5`-TCAATTCCCTCTATTTGGTGGT-3`
aph(3')-IIIa	aph3	5`-(HEX)- CGAGCTGTATGCGGAGTGCATCAGGCTCT-(BHQ1)-3`
	Fap3	5`-AGGGACAATCCGATATGTCG-3`
	Rap3	5`-TGAAGATGAACAAAGCCCTGA-3`
ant(4')-Ia	adD	5`-(HEX)- TGGCTCTCTTGGTCGTCAGACTGATGGGC-(BHQ1)-3`
	FdD	5`-GGATGATGTTAAGGCTATTGGTG-3`
	RdD	5`-CACATCATCTCAATATCCGAATAGG-3`
aac(6')-Ie-aph(2'')-Ia	zBif	5`-(HEX)- AGATTTGCCAGAACATGAATTACACGAGGGCA-(BHQ1)-3`
	FBif	5`-AAGGGCATACCAAAAATCTG-3`
	RBif	5`-CCTTAACATTTGTGGCATTATCAT-3`
